# Editorial: Herbal medicines in women’s lives

**DOI:** 10.3389/fphar.2022.1003241

**Published:** 2022-11-16

**Authors:** Shan-Yu Su

**Affiliations:** Department of Chinese Medicine, China Medical University Hospital, School of Post-Baccalaureate Chinese Medicine, College of Chinese Medicine, China Medical University, Taichung, Taiwan

**Keywords:** herbs, dysmenorrhea, infertility, pregnancy, menopausal syndrome

In many areas, natural products are traditionally used to improve body conditions in several periods of a women’s life, including adolescent, reproductive, and menopausal periods. In the adolescent period, medical herbs are used to enhance growth. At childbearing age, medical herbs are used to treat menstruation problems, decrease vaginal infection, and increase fertility. During pregnancy, medical herbs are used to reduce discomfort and prevent miscarriage. After menopause, medical herbs are used to alleviate symptoms and delay degeneration. All the above-mentioned usages of natural products are considered to improve life quality for women. Herbs used in women’ lives deserve careful study. The articles in this Research Topic involve the use of herbal medicines during childbearing and menopausal periods. The conditions involved dysmenorrhea, endometriosis, infertility, lactation, osteoporosis, and menopausal symptoms.

At child-bearing age, dysmenorrhea affects 45% of women (Iacovides et al., 2015). In several countries and areas with traditional medicines, herbs are commonly used to relieve cramps in primary and secondary dysmenorrhea. In this Research topic, the research of Su et al. discloses the herbs used for dysmenorrhea in Taiwan *via* a field investigation among traditional Chinese medicine pharmacies. Those pharmacies are popular among women in Taiwan. This research team found that Angelica sinensis (Oliv.) Diels and Ligusticum chuanxiong Hort are two important herbs in the prescriptions of Taiwanese pharmacies. Among the etiologies of dysmenorrhea, endometriosis is a common and intractable disease that causes secondary dysmenorrhea (Mehedintu et al., 2014). Several herbs are shown to inhibit the pathogenesis of endometriosis, such as inflammation, proliferation, and angiogenesis (Meresman et al., 2021). Endometriosis patients who take the herbs are also shown to have a lower surgery rate than those who do not take herbs (Su et al., 2014). Zheng et al. provide possible mechanisms of a traditional Chinese medicine formula, the ELeng Capsule. By transcriptomics combined with network analysis, Zheng et al. conclude that the potential mechanisms to treat endometriosis of the Eleng Capsule might include apoptosis and regulating angiogenesis, cytoskeleton, and epithelial-mesenchymal transition.

Fertility is also an important issue during the childbearing age. Zishen Yutai Pills is a famous formula taken by the female population who want to get pregnant in China. Li et al. conducted a metabonomics study to identify enriched pathways for Zishen Yutai Pills in women who were undergoing *in vitro* fertilization. Their results show that Zishen Yutai Pills down-regulated the chemicals of trihexosylceramide, glucosylceramide, and TG, and up-regulated chemicals of PIP3, PIP2, tauroursodeoxycholic acid, L-asparagine, L-glutamic acid, kynurenic acid, 11-deoxycorticosterone, melatonin glucuronide, and hydroxytyrosol. After pregnancy, women face miscellaneous pregnancy-related symptoms. Even during a normal pregnancy, due to the changes in hormones and the growth of the fetus, there are still common symptoms including morning sickness, abdominal bloating, waist pain, edema, varicosity, genitourinary infection, and mood swings (Nazik and Eryilmaz, 2014). However, women are usually careful in taking medicines to relieve the above symptoms because of some famous harmful effects on the fetus caused by medications. Instead, herbal products are popular among pregnant women for treating symptoms. This Research Topic includes a report by Gantner et al., who find that 52.0% of all pregnant women in Zurich suffer from mild mental disorders, but only 1.3% of them took psychoactive medications. They also find that Kalanchoe pinnata (Lam.) Pers, Lavandula angustifolia Mill, and Valeriana officinalis L. were the three herbs that pregnant women used the most. Those herbs were used to reduce stress, restlessness, and sleep disorders. After childbirth, breastfeeding provides health for both mothers and babies. In Tanzania, Millinga et al. conducted a cross-sectional survey using a structured questionnaire to investigate the use of herbal medicines during breastfeeding. They find that 53.8% of breastfeeding mothers used herbs. The most commonly used herbs were Piper nigrum L., and Cucurbita pepo L. We could compare this study with a previous one which was conducted in Taiwan that reported Angelica sinensis (Oliv.) Diels, Tetrapanax papyrifer (Hook.) K. Koch, and Hedysarum polybotrys Hand.-Mazz. as the three most commonly used herbs to promote lactation (Chao et al., 2020). At the same time, Millinga et al. reveal that higher education levels and a low breast milk supply were identified as predictors of the use of herbs among breastfeeding mothers.

There is still an important issue in a women’s life, menopause. Before and after menopause, women face a menopause transition, which is a period that starts from the onset of irregular menstruation or vasomotor symptoms (Gracia and Freeman, 2018). During this time, the majority of women experience uncomfortable symptoms that are collectively called menopausal syndrome, such as vasomotor symptoms, mood changes, and sleep problems (Takahashi and Johnson, 2015). In this Research Topic, there are five articles related to female menopause published. The first one is by Lan et al., which used animal models of rats to generate the conditions of Kidney deficiency and Liver qi stagnation, both of which are pattern conditions happening to menopausal women. The authors find that the combination of Chinese herbal formulae corresponded to the two pattern conditions that corrected menopausal indices the best. The second article by Jalalvand et al. reveals that, in menopausal women, after 10 weeks’ consumption of Elaeagnus angustifolia L., there were increases in thyroid-stimulating hormone, cortisol level, and the ratio of cortisol/dehydroepiandrosterone-Sulfate. However, the level of prolactin decreased. Uncomfortable joints are another issue during or after menopause, especially in Asian women (Haines et al., 2005). The third article is a double-blind, randomized controlled trial conducted in Korea by Kim et al. They report that red ginseng is an effective supplement that reduced the pain score in menopausal women with degenerative osteoarthritis in the hand. Osteoporosis is a severe condition that usually happens in menopausal women and puts them at risk of bone fractures (Li and Wang, 2018). The fourth article is about an herbal formula Shen-sui-tong-zhi formula, which has been used in China for treating musculoskeletal disorders for years. Xu et al. use a mouse model to dig out mechanisms for the bone density-protective effect provided by the Shen-sui-tong-zhi formula. The authors find that the main pathway Shen-sui-tong-zhi formula provides for its osteogenesis was the activation of β–catenin signaling in growth plate chondrocytes. Lastly, the fifth article related to female menopause is a study on Salvia miltiorrhiza, which is an herb having both “Blooding activating” and “Blooding tonifying” effects according to the ethnopharmacological usage in Chinese medicine; moreover, Salvia miltiorrhiza has various pharmacological effects and are applied in various clinical illnesses (Shi et al., 2019; XD et al., 2019). Tseng et al. reveal that among people who used herbal medicine, women used Salvia miltiorrhiza more than males. More precisely, Salvia miltiorrhiza was used mostly in women aged 35–49 years, those were before and during menopausal transition. Moreover, the most common diseases treated by Salvia miltiorrhiza were menopausal disorders combined with general symptoms. The most frequent formulae jointly used with Salvia miltiorrhiza were herbal formulae of Yan-Hu-Suo and Jia-Wei-Xiao-Yao-San.

During the whole life of a women, blood is an important issue. Women often suffer from anemia in many diseases and non-disease conditions, such as fibroids, adenomyosis, and uterine atony after delivery or during menstruation (Khafaga and Goldstein, 2019). women often suffer from dizziness, palpitation, and weakness even when not having anemia. These symptoms are called blood-deficient syndrome in traditional Chinese medicine. Blood-deficient syndrome lowers the quality of life of women. There is a noble medicinal material in Chinese medicine, donkey-hide gelatin, which is not a plant. Instead, it is an Equus asinus-derived medicinal material that is traditionally used to supply blood to the body. To test its efficacy in blood deficiency-related symptoms, Zhang et al. perform a randomized, double-blind, and placebo-controlled clinical trial. Their results show that after taking 6 g of donkey-hide gelatin per day for 2 months, the symptom of dizziness was reduced and the quality of life was improved. Moreover, after 2 months, there were significant differences between the treatment and control groups in hematocrit and red blood cell numbers.

Besides treating illnesses, herbs provide cosmetic purposes. Asian women think white skin is beautiful and cosmetic companies always invent skin-whitening products (Spyropoulou et al., 2020). Asian women also take or apply skin-whitening herbal products. Ko et al. perform a field investigation on skin-whitening herbal prescriptions in Taiwan. They find that Wolfiporia extensa (Peck) Ginns, Glycyrrhiza uralensis Fisch., and Paeonia lactiflora Pall. were the three most frequently sold herbs used for skin-whitening by herbal pharmacies. They have the potential to become commercial products after safety and efficacy tests.

The articles included in this Research Topic provide a view of how herbal medicine plays a role and how herbs weave a whole story in a women’s life. Some of these articles survey detailed mechanisms of herbal medicine, and some of them provided the first glimpse into the phenomenon of using herbal medicines. The doses used in animal models recruited in this Research Topic cover human equivalent doses, therefore, the results could be used as references for clinical application. All the articles in this Research Topic could be the basis of research tomorrow.
